# Conservation tillage facilitates the accumulation of soil organic carbon fractions by affecting the microbial community in an eolian sandy soil

**DOI:** 10.3389/fmicb.2024.1394179

**Published:** 2024-05-31

**Authors:** Yu-mei Li, Yu-ming Wang, Guang-wei Qiu, Hong-jiu Yu, Feng-man Liu, Gen-lin Wang, Yan Duan

**Affiliations:** ^1^Heilongjiang Black Soil Conservation and Utilization Research Institute, Harbin, China; ^2^The Centre for Ion Beam Bioengineering Green Agriculture, Hefei Institutes of Physical Science, Chinese Academy of Sciences, Hefei, China; ^3^Science Island Branch, Graduate School of USTC, Hefei, China; ^4^Keshan Branch of Heilongjiang Academy of Agricultural Sciences, Qiqihar, China

**Keywords:** conservation tillage, particulate organic carbon, mineral-associated organic carbon, microbial community, soil depth

## Abstract

Conservation tillage (CT) is an important agronomic measure that facilitates soil organic carbon (SOC) accumulation by reducing soil disturbance and plant residue mulching, thus increasing crop yields, improving soil fertility and achieving C neutrality. However, our understanding of the microbial mechanism underlying SOC fraction accumulation under different tillage practices is still lacking. Here, a 6-year *in situ* field experiment was carried out to explore the effects of CT and traditional tillage (CK) practices on SOC fractions in an eolian sandy soil. Compared with CK, CT increased the particulate OC (POC) content in the 0–30 cm soil layer and the mineral-associated OC (MAOC) content in the 0–20 cm soil layer. Moreover, tillage type and soil depth had significant influences on the bacterial, fungal and protistan community compositions and structures. The co-occurrence network was divided into 4 ecological modules, and module 1 exhibited significant correlations with the POC and MOC contents. After determining their topological roles, we identified the keystone taxa in the network. The results indicated that the most common bacterial taxa may result in SOC loss due to low C use efficiency, while specific fungal (Cephalotrichum) and protistan (Cercozoa) species could facilitate SOC fraction accumulation by promoting macroaggregate formation and predation. Therefore, the increase in keystone fungi and protists, as well as the reduction in bacteria, drove module 1 community function, which in turn promoted SOC sequestration under CT. These results strengthen our understanding of microbial functions in the accrual of SOC fractions, which contributes to the development of conservation agriculture on the Northeast China Plain.

## 1 Introduction

Soils are the largest carbon (C) pool in the global terrestrial system and contain more than 2,500 Gt of C (Banerjee et al., 2016). Global soil organic carbon (SOC) dynamics immensely influence soil productivity, greenhouse gas emissions and C neutrality (Tang et al., 2019; Duan et al., 2023). In agroecosystems, tillage practices are regarded as crucial agronomic regimes that mediate SOC sequestration and depletion processes (Topa et al., 2021). Traditional agricultural practices involve frequent tillage to accomplish achieve high crop productivity (Zhang et al., 2019). However, high-intensity tillage not only decreases crop productivity but also leads to reduced soil fertility and sustainability and results in the loss of other agroecosystem services, causing soil erosion, water shortages or biodiversity decline (Raus et al., 2016). Hence, abundant attempts have been made to transition from traditional tillage to conservation tillage (CT) to increase the soil C stock of agroecosystems in recent years. In particular, CT practices, such as no-tillage or reduced-tillage, may minimize the degree and frequency of tillage passes and maintain an adequate soil surface covered with residues to reduce soil physical disturbance and increase the soil C sink capacity (Pearsons et al., 2023). Nevertheless, contrasting tillage practices cause changes in resource availability in the topsoil and subsoil, which leads to differences in SOC formation (Angers and Eriksen-Hamel, 2008). Therefore, elucidating the SOC sequestration mechanisms that occur under different tillage practices and at different soil depths is crucial for maintaining soil health and facilitating agroecosystem services.

Overall, the input of exogenous organic materials (i.e., manure and crop residues) is an essential prerequisite for SOC accumulation (Lehmann and Kleber, 2015). Many expert researchers have confirmed that the mechanisms underlying SOC accumulation are commonly attributed to physical protection by aggregates and chemical stabilization by soil minerals (Six et al., 2004). Correspondingly, semidecomposed exogenous large organic biopolymers are readily encapsulated by aggregates and form particulate organic carbon (POC); as biopolymers further decompose, the C monomers tend to be adsorbed by mineral surfaces and become mineral-associated organic carbon (MAOC) (Bastian et al., 2009; Herath et al., 2014). There is nearly a consensus regarding the disruption of topsoil aggregates due to frequent tillage, which leads to the loss of POC (Hewins et al., 2017). These conclusions also reflect the potential of using CT to increase SOC sequestration in agroecosystems. Several previous studies also suggested that traditional tillage practices involving straw return can transport crop residues to the subsoil, thus contributing to the accumulation of SOC in the subsoil to some extent (Zhang et al., 2013). This contradiction also illustrates the complexity of enhancing soil fertility through tillage practices. Achankeng and Cornelis (2023) found that climate, soil texture, rotation pattern, and crop type are all crucial factors to be considered under different tillage treatments in a meta-analysis, which increases the challenge for researchers in optimizing tillage practices. Therefore, to date, we still lack a comprehensive understanding of the direct associations between tillage practices and SOC fractions.

Soil microorganisms play an important role in SOC formation (Cotrufo et al., 2013; Sarker et al., 2018). Generally, when plant residues are applied, soil animal- and meso-fauna-driven fragmentation constitute the first stage of straw degradation (Gessner et al., 2010). Subsequently, bacteria and fungi successively regulate further degradation processes due to changes in C and N availability in the substrate (Wang et al., 2021). In the early stage of decomposition, adequate amounts of labile straw C and nitrogen can sustain bacterial proliferation (Huang et al., 2017). With continuous plant decomposition, the microbial community synchronously undergoes succession. Fungi may be the dominant decomposers due to their potent ability to utilize recalcitrant straw components (Clemmensen et al., 2015). Therefore, many scholars consider the ratio of bacteria to fungi to be an important indicator of the straw decomposition process (Li J. W. et al., 2020; Zhao et al., 2021). Moreover, microbial diversity and module community was the key drivers of SOC turnover. Previous study found that bacterial, fungal protistan richness was significantly correlated with carbon use efficiency, microbial biomass carbon, microbial respiration and growth rate, which changed the SOC turnover process (Ma et al., 2024). Soil microbial module community also played an important role in influencing SOC. Numerous studies have demonstrated that the core microbial module community was involved in maintaining the stability of soil microbial function, promoting soil nutrient cycling and SOC accumulation; while the keystone species were the core to achieve them (Shi et al., 2020; Jiao et al., 2022). Therefore, keystone taxa-driven the changes of microbial module communities and diversity are pivotal factors leading to SOC turnover. In particular, soil bacterial and fungal communities are extremely sensitive to tillage practices. Li Y. et al. (2020) reported that, compared with traditional tillage, minimum tillage increases fungal biomass and bacterial diversity, which may further influence residue decomposition. However, as major members of the soil microbiome, protists drive plant residue decomposition, and microbial community regulation has rarely been included in microbiome analyses associated with SOC fraction turnover (Geisen and Bonkowski, 2018). Specific protozoan taxa participate in aggregate formation and SOC turnover. According to the report of Pellegrino et al. (2021), CT increased the abundance of Alveolata and Cercozoa, which contributed to SOC accumulation by reshaping soil aggregates. Additionally, the top-down control of protists in the soil microfood web demonstrated great potential for influencing SOC turnover (Gao et al., 2019). Therefore, a thorough empirical understanding of the microbial roles (including bacterial, fungal and protist roles) in SOC fraction sequestration under different tillage practices has not been achieved.

To bridge these gaps, eolian sandy soil located in the Northeast Plain, the largest grain-producing area in China, was selected as the research object. In recent years, the Northeast Plain has been facing continuous depletion of SOC stocks since the 1980s, when straw return was widely implemented (Wang et al., 2018). Therefore, we conducted a 5-year *in situ* field experiment to reveal the effect of tillage practices on the microbial community, SOC fraction and maize yield at different soil depths. The soil samples were collected from the 0–50 cm soil profile under CT and traditional tillage practices. In this study, we determined the SOC fraction content and microbial community and attempted to explain the potential relationships between them. We hypothesized that (1) the SOC fraction content and microbial traits exhibit distinct responses to traditional and CT practices at different depths and that (2) specific microbial taxa may be involved in the turnover of SOC fractions.

## 2 Materials and methods

### 2.1 Experimental site

The experimental field was located in Dulbert Mongolian Autonomous County (46°54′N, 124°26′E), Daqing city, Heilongjiang Province, which has a semiarid monsoon continental climate. The mean annual precipitation and temperature are 400 mm and 5.6°C, respectively. According to the USDA soil taxonomy, the soils in the area are carbonate meadow soils. The basic nutrient contents of the soil before the experiment were as follows: 0.53 g kg^−1^ total nitrogen; 60.95 mg kg^−1^ alkali-hydrolyzable nitrogen; 60.22 mg kg^−1^ available phosphorus; 44.51 mg kg^−1^ available potassium; and 9.52 g kg^−1^ SOC with a pH of 5.56. The cropping system used was single spring maize (*Zea mays* L.).

### 2.2 Field trial design and soil sampling

The experimental trial was set up in 2017 in accordance with a randomized complete block design with three replicates. Each field plot was covering an area of 64 m^2^ (4 m × 16 m). Before the experiment, all the plots were treated with N-P-K fertilizers, and straw was removed via shallow tillage to 25 cm. Traditional tillage (CK): the plots were plowed with large machinery, and a five-share turning plow tilled the soil to 25 cm after harvest at October (amount of maize stover return about 7,200 kg ha^−1^); and CT: no-tillage with 100% straw mulch after harvest at October (amount of maize stover mulch about 7,600 kg ha^−1^). A straw crusher was used to crush the straw into fragments with lengths less than 10 cm before mulching. Except for the field surface drilling of maize in October, the no-tillage plots remained undisturbed, and maize straw was evenly distributed over the field surface after harvest every year. Chemical fertilizers were applied in May, and the N-P_2_O_5_-K_2_O application rates ranged from 180–115–75 kg hm^−2^. All the other normal management practices were consistent between the treatments during the experiment.

Soil profiles were excavated at a depth of 50 cm in October 2022 in each replicate plot. Soil samples were collected at depths of 0–10, 10–20, 20–30, 30–40, and 40–50 cm. Three soil samples were collected from each plot. The three soil samples were placed into the same sterile plastic bag and mixed to create one composite sample. All the samples were immediately transported to the laboratory in an incubator with ice packs. Each soil sample was divided into two parts: one part was stored at −80°C for DNA extraction, and the remaining part was air-dried for use in the additional chemical analyses. The basic chemical properties of the soils under different tillage practices and at different depths in 2022 are shown in Supplementary Figure S1.

### 2.3 Basic soil properties, SOC fractionation, and soil aggregate isolation

The basic chemical properties of the soil were measured using the method described by Lu (2000). The soil pH was measured at a 1:2.5 soil: water ratio for 30 min. The SOC concentration was determined using K_2_Cr_2_O_7_ digestion, and total nitrogen was determined by the Kjeldahl method. Available phosphorus and potassium were determined using molybdenum blue colorimetric and flame photometry methods, respectively.

SOC fractionation was determined using a method described by Yu et al. (2017). First, the SOC was further fractionated into POC and MAOC. Generally, 5.0 g (dry weight) of soil was dispersed by adding 30 mL of 0.5% sodium hexametaphosphate solution and centrifuging at 200 r min^−1^ for 18 h. Thereafter, the POC and MAOC fractions were obtained by passing the samples through 53-μm filters. All the fractions were dried (50°C), and POC and MAOC were measured using K_2_Cr_2_O_7_ digestion.

The wet sieving method was used to separate the water-stable aggregates (Six et al., 2002). First, the soil samples were gently broken apart into small pieces along natural break points, and the fragmented samples were subsequently placed on top of a 0.25-mm sieve and soaked in deionized water for 5 min. The samples were subsequently separated at an amplitude of 3 cm and a frequency of 30 cycles per min for a duration of 2 min by a wet-sieving apparatus. Afterward, the aggregate subsamples above each sieve were obtained as follows: macroaggregates (>0.25 mm), microaggregates (0.053–0.25 mm), and silt and clay fractions (<0.053 mm). After wet sieving, all the aggregates were immediately oven-dried at 60°C and weighed.

The mean weight diameter (MWD) was used to describe the aggregate stability and was calculated by the following formula:


MWD=∑Xi∗Wi

where Xi represents the average diameter of each aggregate size and Wi represents the proportion of each aggregate weight relative to the total sample weight after wet sieving. The upper limit of the macroaggregate diameter was 2 mm.

### 2.4 DNA extraction and 16S, ITS and 18S amplification and sequencing

Total DNA was extracted from 0.5 g of soil using a Fast DNA Spin Kit for Soil (MP Biomedicals, CA, United States) in accordance with the manufacturer’s instructions. Each treatment contained three replicates. The extracted DNA samples were stored at −80°C for molecular analysis.

High-throughput sequencing was performed using the Illumina MiSeq sequencing platform (Illumina, Inc.). Both the forward and reverse primers were tagged with adapter and linker sequences, and 8-bp barcode oligonucleotides were added to distinguish the amplicons derived from different soil samples.

The primers *515F (5′-GTGCCAGCMGCCGCGGTAA-3′)* and *907R (5′-CCGTCAATTCMTTTRAGTTT-3′)* were chosen to amplify the 16S rRNA genes in the V4–V5 hypervariable region. PCR was conducted in a 50-μL reaction mixture containing 27 μL of ddH_2_O, 2 μL (5 μM) of each forward/reverse primer, 2.5 μL (10 ng) of template DNA, 5 μL (2.5 mM) of deoxynucleoside triphosphates, 10 μL of 5× Fastpfu buffer, 0.5 μL of bovine serum albumin, and 1 μL of TransStart Fastpfu polymerase (TransGen, Beijing, China). The PCR procedure was 94°C for 5 min; 30 cycles of 94°C for 30 s, 52°C for 30 s and 72°C for 30 s, followed by 72°C for 10 min (Biddle et al., 2008).

The fungal ITS1 region was amplified using the primer pair *ITS1F (CTTGGTCATTTAGAGGAAGTAA)/ITS2 (GCTGCGTTCTTCATCGATGC)*. The 50-μL reaction mixture contained 1 μL (30 ng) of DNA, 4 μL (1 μM) each of forward/reverse primer, 25 μL of PCR Master Mix, and 16 μL of ddH_2_O. PCR amplification was conducted at 98°C for 3 min, followed by 30 cycles of 98°C for 45 s, 55°C for 45 s, and 72°C for 45 s, with a final extension at 72°C for 7 min (Ghannoum et al., 2010).

The eukaryotic V4 region was amplified using the primer pair V4_1f *(CCAGCASCYGCGGTAATWCC)/TAReukREV3 (ACTTTCGTTCTTGATYRA)*. PCR was performed in a 20 μL volume consisting of 4 μL of 5× reaction buffer, 2 μL of dNTPs (2.5 mM), 0.8 μL of each primer (10 μM), 0.4 μL of FastPfu Polymerase, 10 ng of DNA template, and ddH_2_O to reach the final volume. PCR amplification was conducted at 95°C for 5 min, followed by 30 cycles of 95°C for 30 s, 55°C for 30 s, and 72°C for 45 s, with a final extension at 72°C for 10 min. To construct the protistan amplicon sequence variant (ASV) table, we removed sequences belonging to Rhodophyta, Streptophyta, Metazoa, and Fungi (Stoeck et al., 2010).

Raw Illumina amplicon reads were processed using the QIIME2 Core 2019.7 distribution. The Divisive Amplicon Denoising Algorithm (DADA2) pipeline implemented in the QIIME 2 platform was used to conduct sequence quality control, which included quality filtering reads, denoising reads, merging forward and reverse reads, removing chimeric reads, and assigning reads to ASVs. The Silva 138 Bacterial 16S rRNA gene database, the UNITE Fungal ITS database, and the Protist Ribosomal Reference (PR2) database v4.14.0 were used to classify the representative sequences of ASVs (Ghannoum et al., 2010). All singletons and nonfungal ASVs were removed, and each sample was rarefied to 30,000, 36,000, and 44,000 sequences for the bacterial, fungal and eukaryotic diversity analysis. The alpha diversity and Bray–Curtis distances for principal coordinate analysis of the soil microbial community were calculated after all the samples were rarefied to the same sequencing depth.

### 2.5 Statistical analysis

Crop yield and soil biochemical and other relevant properties under different tillage practices were subjected to the chi-square test for independence of variance. Significant differences were determined by one-way analysis of variance (ANOVA) based on the *post hoc* Tukey test at the 5% level. Prior to ANOVA, the normality and homogeneity of variance were tested by the Kolmogorov–Smirnov test and Levene’s test, respectively. If normality was not met, log or square-root transformation was carried out. One-way ANOVA was performed using SPSS 21.0 (SPSS, Inc., Chicago, IL, United States).

Principal component analysis (PCA) was used to determine and evaluate the changes in the community structure of the soil microbiome via the R (ver 4.2.3) package “vegan.” To characterize the patterns of soil microbial interactions, we constructed a co-occurrence network with the “igraph” and “WGCNA” R packages. We constructed microbial networks using bacteria, fungi and protists with relative abundances greater than 0.01%; screened nodes with Pearson’s correlations greater than 0.6 and *p* < 0.05; performed modular analysis based on the connectivity between nodes; visualized the network using Gephi (ver. 0.9.2); and calculated information on network topological features. The within-cluster connectivity (*Zi*) and among-cluster connectivity (*Pi*) of different clusters were calculated using the R packages “reshape2,” “igraph,” “ggrepel,” “dplyr,” and “Rcpp” and filtered for peripherals (*Zi* ≤ 2.5, *Pi* ≤ 0.62), connectors (*Zi* ≤ 2.5, *Pi* > 0.62), cluster hubs (*Zi* > 2.5, *Pi* ≤ 0.62), and network hubs (*Zi* > 2.5, *Pi* > 0.62) (Deng et al., 2012). ASVs in module hubs, connectors and network hubs may be regarded as the microbial keystone taxa of network systems (Deng et al., 2012). An interactive platform “Gephi” (default parameters set) was used to identify the modules (ecological clusters) of soil taxa strongly interacting with each other.

Linear regressions between SOC fractions and the microbial community modules were conducted to determine the relationships between microbial communities and SOC fraction contents using Origin 2018. The microbial module community variation data were quantified by the PCA 1 axis. Heatmaps were constructed to reveal the potential associations between keystone taxa richness and SOC fraction content via the “heatmap.2” function in the R package “ggplots.”

## 3 Results

### 3.1 Crop yields, aggregate size distributions, and SOC fractions

**Figure 1:**
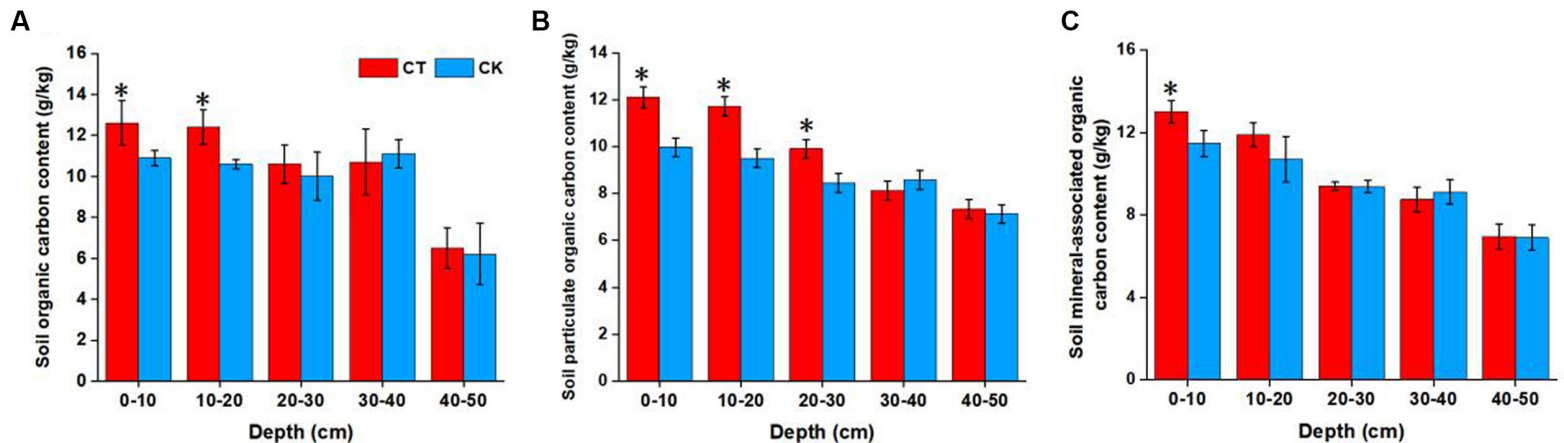
Soil organic carbon **(A)**, particulate organic carbon **(B)**, and mineral-associated organic carbon **(C)** contents at various depths under different soil tillage treatments. *p < 0.05; CT, conservation tillage; CK, traditional tillage.

Overall, CT had a positive effect on crop yield and SOC fraction content. After 5 consecutive years of different tillage practices, the maize yield, aggregate stability and SOC fraction content changed significantly (Supplementary Figures S2, S3); Figure 1). However, no significant changes were found before 2021. Compared with those in CK, the maize yields in CT significantly increased in 2021 and 2022 (Supplementary Figure S2, *p* < 0.05).

Different tillage practices changed the aggregate size distribution and stability (Supplementary Figure S3). CT significantly increased the proportion of macroaggregates at the 0–30 cm soil depth (*p* < 0.05) and decreased the proportion of microaggregates at the 0–20 cm soil depth (*p* < 0.05). However, no changes were observed in the silt and clay fractions under the different treatments. Additionally, the MWD was greater under CT than CK at 0–30 cm depth, while there were no significant changes at 40–50 cm depth.

SOC, POC, and MAOC contents were also affected by tillage practice (Figure 1). Generally, CT significantly increased the SOC, POC, and MAOC contents in the topsoil (0–20 cm layer, *p* < 0.05, except for MAOC in the 10–20 cm layer). Moreover, the POC content was significantly greater under CT than CK. No significant changes were observed in the 30–50 cm layer under the different tillage practices.

### 3.2 Microbial community, co-occurrence network, and keystone taxa

PCA was used to evaluate the changes in the soil microbial community under the different tillage practices (Supplementary Figures S4, S5). The results indicated that the soil communities changed significantly under the different tillage practices and at different soil depths (except for tillage practices on the protistan community). Generally, the effect of soil depth on microbial communities was observed mainly along the PCA 1 axis, while the effect of tillage practices on microbial communities was observed mainly along the PCA 2 axis.

Although soil microbial community compositions were changed after different tillage practices and depths, Proteobacteria, Acidobacteriota, Actinobacteriota, and Gemmatimonadota were the main phyla of bacteria, contributing more than 60% of the total bacterial abundance (Supplementary Figure S6A). Generally, with the increase of depth, the relative abundance of Proteobacteria decreased gradually. Ascmycota, Basidiomycota, and Mortierellomycota were the main phyla of fungi, contributing almost 80% of the total fungal abundance (Supplementary Figure S6B); while protist were composed mainly of Intramacronucleata, Cercozoa, Chlorophyta, and Apicomplexa (Supplementary Figure S6C).

**Figure 2:**
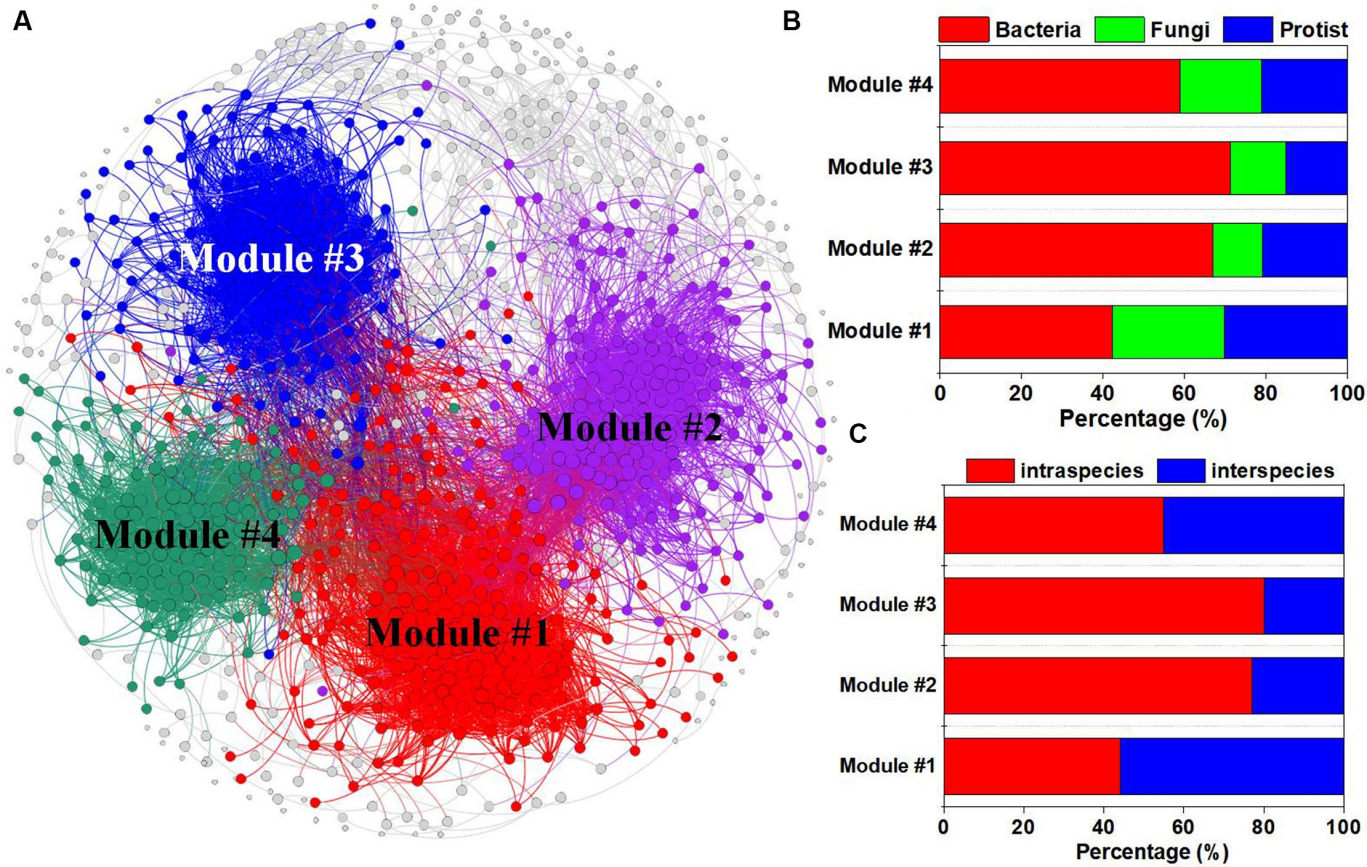
Co-occurrence network analysis of bacterial, fungal and protistan ASVs under different tillage practices and at different soil depths. **(A)** Multitrophic network including multiple ecological modules. The colors of the nodes represent different ecological modules; the percentages of bacterial, fungal and protistan ASVs **(B)**; and the intraspecies and interspecies relationships **(C)** in each module.

A co-occurrence network was constructed to reveal the connections between specific microbial species (Figure 2). We found four dominant ecological modules (Figure 2A). Modules 1, 2, 3, and 4 contained 225, 182, 160, and 124 nodes, respectively. Among the four modules, protists and fungi accounted for the greatest proportion of microbial species in module 1, while bacteria accounted for the highest proportion of microbial species in module 3 (Figure 2B). Additionally, the percentage of intraspecies edges was greater in module 3 than in the other modules, while module 1 contained more interspecies edges than did the other modules (Figure 2C).

**Figure 3:**
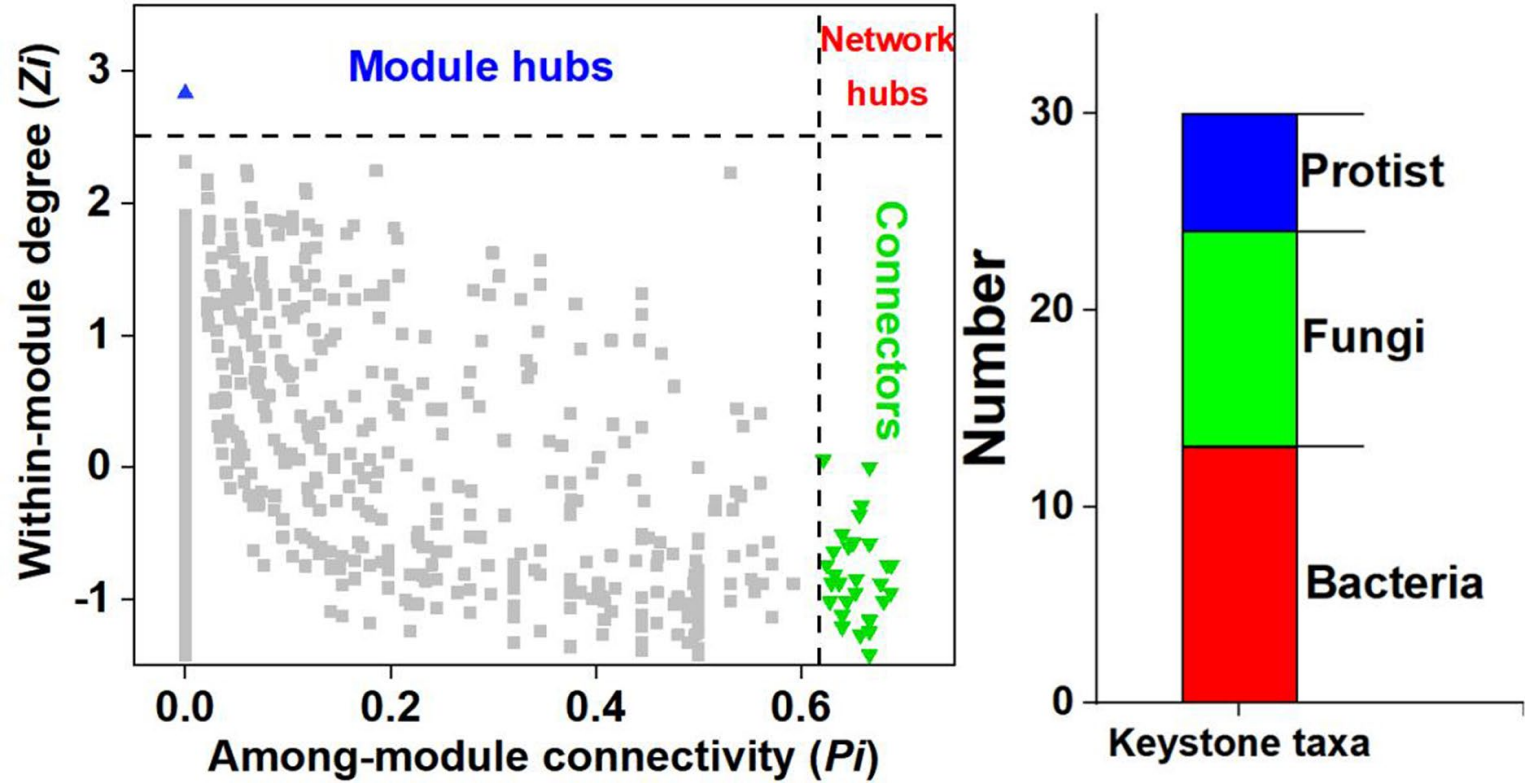
ZP plot showing the distribution of ASVs based on their module-based topological roles. The topological role of each ASV was determined according to the scatter plot of within-module connectivity (Z) and among-module connectivity (P).

*ZP* plots were constructed to identify the topological roles of each node in the co-occurrence network. A total of 30 microbial taxa (including 13 bacteria, 11 fungi and 6 protists) were detected as keystone species (Figure 3). Nineteen keystone taxa belonged to module 1, and modules 2 and 3 each contained 2 keystone taxa. The information for the selected keystone taxa is displayed in Supplementary Table S1. The bacterial keystone species mainly belonged to Proteobacteria (7 taxa), the fungal keystone species mainly belonged to Ascomycota (6 taxa), and the protist keystone species mainly belonged to Cercozoa (2 taxa).

### 3.3 Relationships between microbial traits and SOC fractions

**Figure 4:**
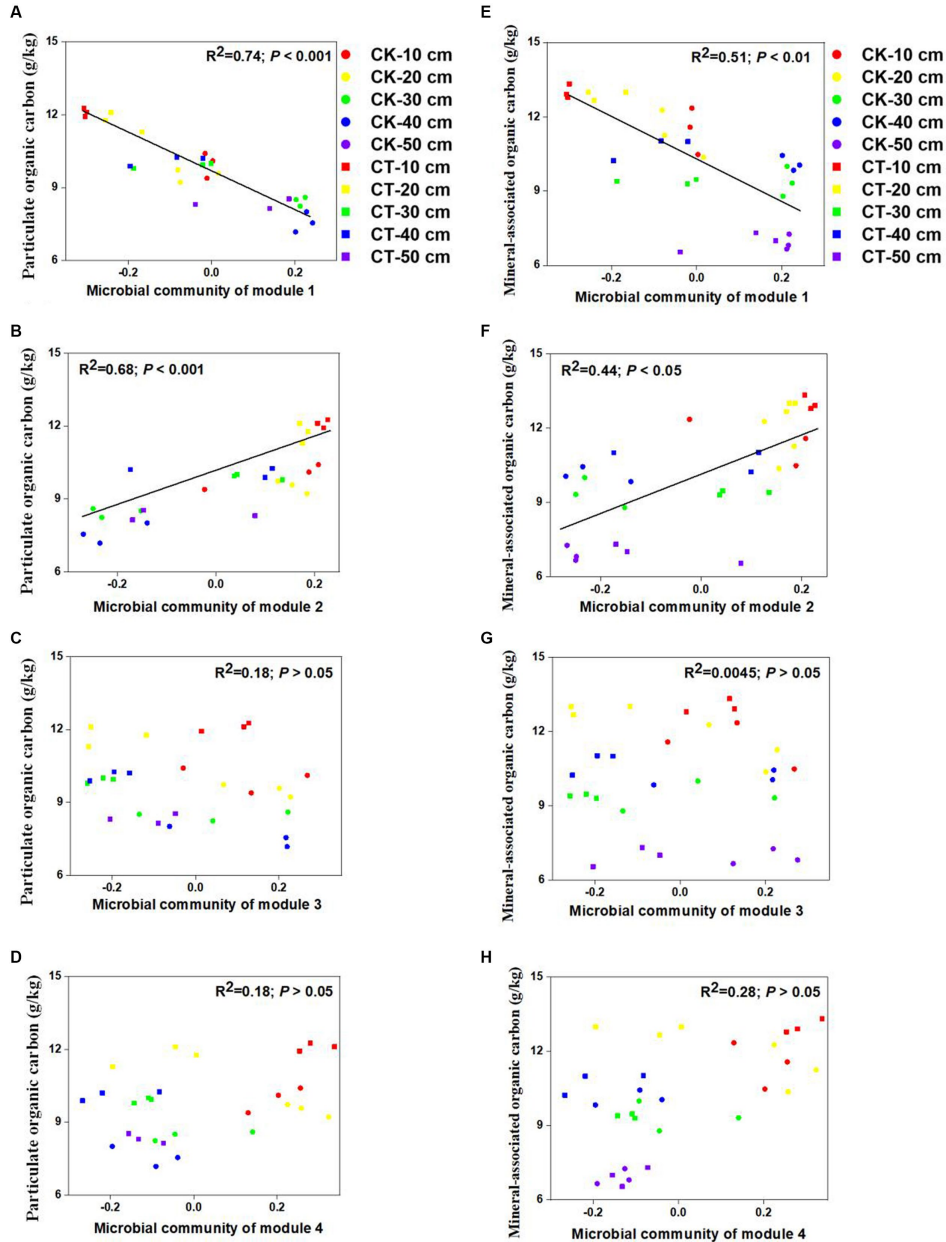
Links between the soil community of each module within the co-occurrence network and the SOC fraction content. The links between soil microbial community of module 1-4 with particulate organic carbon were sown in A-D; the links between soil microbial community of module 1-4 with mineral-associated organic carbon were sown in E-H.

To determine the potential relationships between the microbial communities of specific modules and SOC fractions, we constructed correlations between the microbial community module connections and the SOC fractions. Figure 4 shows that there were significant correlations between microbial module communities and POC content (*R*^2^ = 0.74 for module 1 and *R*^2^ = 0.68 for module 2) and MAOC content (*R*^2^ = 0.51 for module 1 and *R*^2^ = 0.44 for module 2). However, no significant associations were detected between the SOC fractions and the other microbial module communities.

**Figure 5:**
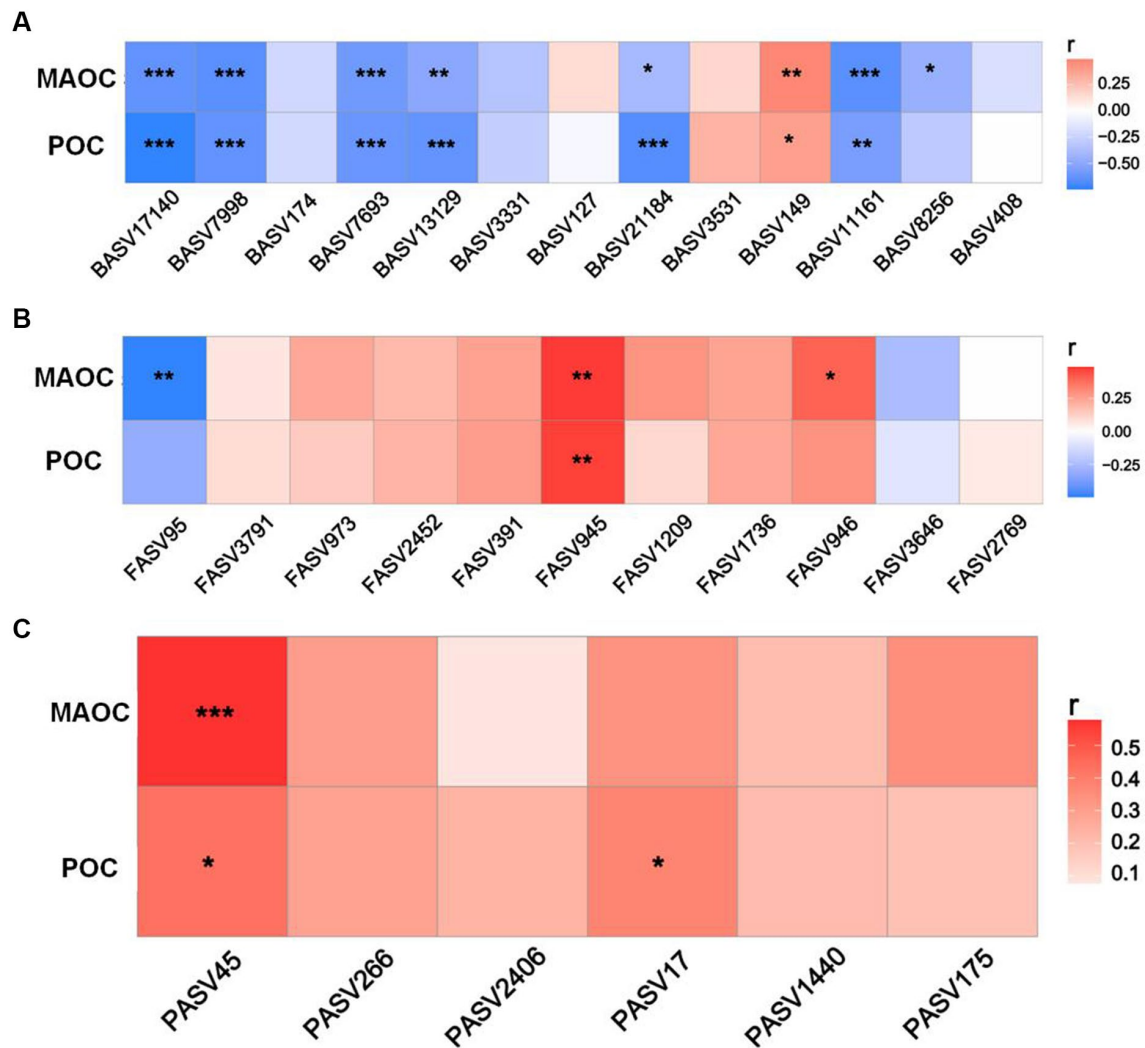
Relationships of SOC fraction contents with the richness of bacterial **(A)**, fungal **(B)**, and protistan **(C)** keystone species.

Heatmaps revealed close associations between the richness of keystone taxa and SOC fractions (Figure 5). Overall, the bacterial richness demonstrated significant negative correlations with the POC and MAOC contents (except for BASV 147) (Figure 5A). There were significant positive correlations between FASV945 richness and SOC fraction contents (POC and MAOC), as well as between FASV945 richness and MAOC content. FASV95 richness was negatively correlated with MAOC content (Figure 5B). Moreover, the richness of PASV45 and PASV17 was positively correlated with the POC content, and the richness of PASV45 was also positively correlated with the MAOC content (Figure 5C).

In the present study, we selected keystone species that were significantly associated with the SOC fraction content for further analysis. The richness of keystone taxa was sensitive to the different tillage practices (Supplementary Figure S7). Compared with CT, CK increased the richness of bacterial keystone taxa by 21.51–520.75%. The richness of BASV8256 decreased by 32.15% under CK compared with that under CT (Supplementary Figure S7A). Compared with CK, CT increased the richness of FASV945 and FASV95 by 58.81 and 42.81%, respectively, and decreased the richness of FASV946 by 82.61%. In addition, compared to that under CK, PASV17 and PASV45 richness increased by 97.79 and 12.97%, respectively, under CT (Supplementary Figure S7B).

## 4 Discussion

CT has been considered a sustainable technique for properly managing soil and hence maintaining agroecosystem services by minimizing tillage operations to effectively avoid water infiltration and erosion (Müller et al., 2009). In this study, we compared crop yields, SOC fraction contents and microbial traits under different tillage practices and investigated the relationships between SOC fractions and microbial functions. The results of this research strengthen our understanding of SOC accumulation under different tillage practices on the Northeast China Plain.

### 4.1 Response of SOC fractions and maize yields to different tillage practices

The effect of tillage practices on crop yields has been studied frequently; however, no consistent conclusions have been drawn. It was reported that no- or minimum-tillage practices led to a 0–30% reduction in yields in Europe, which was affected by crop type, tillage technique, soil texture and crop rotation (Alaoui et al., 2020). Another study revealed that shallow tillage (8 cm strip depth) achieved the greatest yields (Licht and Al-Kaisi, 2005). This is mainly because shallow tillage can be a neutral solution to the problem of late seed emergence due to no-tillage by reducing soil disturbance (Araya et al., 2021). Notably, based on a 17-year experiment, CT practices increased maize yields by 12.2 to 20.1% (Ren et al., 2024), which was consistent with our results (Supplementary Figure S2). This can be explained partly by the fact that the CT method is generally implemented with straw residue left on topsoil while minimizing soil disturbance, which favors soil nutrient accumulation, moisture retention and temperature increase, resulting in faster seed emergence (Licht and Al-Kaisi, 2005). In summary, although optimizing tillage practices requires consideration of factors such as crop rotation and soil texture, CT increases crop yield in maize monoculture systems in the eolian sandy soil of the Northeast China Plain.

SOC is the key to soil fertility and is sensitive to changes in tillage practices (Figure 1). Lehmann and Kleber (2015) proposed a soil continuum model indicating that the input of exogenous organic materials (such as plant residues) is a prerequisite for SOC accumulation. Thus, the finding that CT can improve topsoil OC fractions is no surprise. However, the POC and MOC contents were not consistent. No-tillage practices improved the POC content in the 0–30 cm soil layer. Undecomposed and semidecomposed plant residues are the “core” of POC, which is encapsulated by macroaggregates (Samson et al., 2020). Therefore, the formation of macroaggregates and the accumulation of POC are generally complementary. The data on the distribution of aggregate sizes in the present study also validate this view (Supplementary Figure S3). An increased proportion of macroaggregates provides physical protection for POC and thus favors POC accumulation. CK practices disturb the physical structure at 0–25 cm soil depths through frequent plowing, thus destroying the formation of macroaggregate structures (Jat et al., 2019).

When the large biopolymers in the residues further decomposed, the small biopolymers and C monomers (such as root exudates and microbial necromass) can be adsorbed to the soil mineral surface and become MAOC (Lehmann and Kleber, 2015). In addition to straw return, microbial and maize biomass are important factors that cannot be ignored. As a supplementary exogenous C source in the soil, straw inevitably increases MAOC content after further degradation of residues (Vogel et al., 2014). Posteriorly, CT decreases the soil structure distribution and increases the soil density, which promotes the growth of crop roots to a certain extent (Shi et al., 2012). In addition, the crop yield data imply a well-developed root system that has secreted more organic matter (Van den Putte et al., 2010). With respect to microbial biomass, minimum tillage and residue retention increase the soil microbial population size due to the adequate energy supply and appropriate stoichiometry and result in MAOC sequestration in topsoil under CT practices (Ren et al., 2024). In summary, soil microbiomes play an irreplaceable role in straw degradation and SOC turnover. Therefore, revealing the microbial mechanisms responsible for SOC fraction accumulation under different tillage practices is crucial for improving soil fertility.

### 4.2 Microbial keystone species-driven SOC fraction sequestration by regulating specific module community functions

CT changed the soil microbial diversity and community, which subsequently affected SOC formation and accumulation. We found that soil depth affected the soil bacterial, fungal and protistan communities much more than did tillage practice (Supplementary Figure S4). This is partly because the soil layer has the greatest influence on the changes in nutrient accessibility and availability rather than tillage practices (Kong et al., 2011). As soil depth increases, nutrient pools decrease, and mineral protection increases, leading to increased difficulty in nutrient acquisition by soil microorganisms (Modak et al., 2019). As a result, oligotrophs may become the dominant species in the community.

Microbial communities were classified into different functional modules by identifying soil taxa strongly interacting with each other (Figure 2), which can indicate important ecological processes, different niches, and habitat preferences. Each module in a network is considered a functional unit that conducts an identifiable task (Chen et al., 2019). In the present study, strong relationships were observed between the SOC fractions and the microbial community in modules 1 and 2, which indicated the potential function of SOC turnover (Figure 4). A previous study demonstrated that microbial keystone species have great explanatory power in terms of network (module) structure and function (Delgado-Baquerizo et al., 2018). Thus, it is necessary to explore the function of keystone species within modules 1 and 2. However, more than two-thirds of the identified keystone species belong to module 1 communities (19/30), while only two keystone species (2/30) belong to module 2 communities (Supplementary Table S1). Therefore, it is more meaningful to study the function of keystone species-driven module communities on SOC turnover in module 1 community.

Microbial keystone taxa also exhibited significant correlations with SOC fractions (Figure 5). Among the bacteria, Rhizobiales_Incertae_Sedis (BASV7693) and Reyranellaceae (BASV11161) within module 1 were identified as Proteobacteria, which encompasses typical copiotrophs with low C use efficiency that leads to straw C loss (Dove et al., 2021). A previous study demonstrated that Gaiellaceae (BASV7998) richness is recognized as an indicator of the carbon-to-nitrogen ratio due to its ability to utilize labile C (Duan et al., 2021). Therefore, the increase in the above bacterial taxa indicated negative effects on straw-derived OC accumulation. Moreover, only the Sphingomonadaceae richness exhibited a positive association with the POC and MAOC contents. This was likely because Sphingomonadaceae can consume various C sources and become major exopolysaccharide contributors, which provide a source for MAOC formation (Lan et al., 2022). Compared with bacteria, fungi generally exhibit greater C use efficiency and straw decomposition ability (Clemmensen et al., 2015). The richness of Cephalotrichum (FASV945) and Herpotrichiellaceae (FASV946) in module 1 was conducive to POC and MAOC sequestration. As typical saprophytic fungi, Cephalotrichum and Herpotrichiellaceae are often considered to play roles in straw degradation, pathogen control, and crop growth promotion and are considered important indicators of soil health (Zhang et al., 2022). Zhang et al. (2023) indicated that Cephalotrichum was enriched after straw addition and promoted straw decomposition and SOC accumulation in saline-alkaline soils. Most Cephalotrichum species are known for their saprotrophic function in decomposing plant materials (Woudenberg et al., 2017), corresponding with our results that the Cephalotrichum played a dominant role in POC and MAOC sequestration. In addition, higher abundance of Cephalotrichum led to the higher fungal diversity (Wang et al., 2023). Jin et al. (2022) found that Cephalotrichum exerted significant inhibitory effects on several pathogenic bacteria. Due to these strong abilities, Cephalotrichum abundance was also considered as the biomarker of soil health. Therefore, we speculated that the increase in the abundances of Cephalotrichum can accelerate straw degradation and that the early and late products favor the formation of POC and MAOC, respectively.

In addition, protists can influence SOC accumulation through direct or indirect effects (Pellegrino et al., 2021). Our results revealed that Cercozoa (PASV45 in module 1) may be a possible participant that was significantly positively correlated with POC (*p* < 0.05) and MAOC (*p* < 0.001). Pellegrino et al. (2021) reported that CT practices increase the abundance of Cercozoa partly via several abiotic factors, such as soil moisture, clay content and N availability. As expected, Cercozoa are important consumers of straw residues, which promotes the fragmentation of straw to facilitate further decomposition and is a prerequisite for organic carbon accumulation (Gessner et al., 2010). These authors also indicated that Cercozoa was the keystone taxon in macroaggregates and was positively correlated with SOC by promoting residue decomposition (Delgado-Baquerizo et al., 2020). Moreover, Cercozoa was also thought to have been an important driving force in the formation of macroaggregates by reshaping the pore sizes in the soil (Berisso et al., 2012). Therefore, according to the theory of interaction between SOC and aggregate structure, Cercozoa-driven straw fragments can be encapsulated by aggregates and become the core of aggregate formation. The formation of aggregates provides physical protection for POC, which is conducive to POC accumulation. This conclusion is also consistent with previous reports that Cercozoa are crucial microorganisms in macroaggregate taking part to long-term sequestration and storage of SOC (Pellegrino et al., 2021). Another study indicated that Cercozoa species exhibited the highest numbers of links with bacteria and fungi through the construction of co-occurrence networks, which implied that they were potentially vital to soil food webs (microbiome predation) (Kou et al., 2020). Cercozoa are phagotrophs that may consume Acidobacteria, Proteobacteria, and Ascomycota and consequently increase microbe-derived C. Furthermore, Ma et al. (2024) confirmed that protozoa can regulate microbial carbon use efficiency and SOC formation by regulating fungal, bacterial and keystone module communities through structural equation model analysis. Among these factors, the Cercozoa-driven protozoan community was the most influential factor. Accordingly, Cercozoa mediated POC and MAOC accumulation, mainly through macroaggregate formation and microbial necromass supply.

After comparing the richness of the selected keystone species, our results showed that C-accumulating microbes were enriched under CT. Specifically, compared with CK, CT decreased the abundances of most keystone bacterial taxa and increased the abundances of specific fungal and protistan species in module 1 of the network, which promoted the sequestration of SOC fractions by straw degradation, aggregate formation and predation effects. As a consequence, soil bacteria, fungi and protistan taxa all participate in SOC turnover under different tillage practices, while the appointed keystone species-driven community of module 1 facilitated POC and MOC accumulation under CT.

## 5 Conclusion

In this study, we demonstrated the associations between microbial keystone taxa and SOC fractions under different tillage practices. Compared with CK, continuous 6-year CT significantly increased maize yields, aggregate stability, and POC (0–30 cm) and MAOC (0–20 cm) contents. Tillage practice and soil depth both influence bacterial, fungal and protistan communities, which might change the turnover of SOC fractions. The co-occurrence network indicated that the connectivity of module 1 was significantly related to POC and MAOC contents CT increased the richness of specific fungal (Cephalotrichum) and protistan (Cercozoa) species and thus promoted SOC fraction accumulation through straw degradation, macroaggregate formation and predation effects. The selected bacterial taxa were enriched in the CK treatment and resulted in SOC loss due to low C use efficiency. Taken together, our results revealed that stimulating the function of keystone taxa can drive the function of the module 1 community in SOC accumulation under CT practices, which is beneficial for maintaining soil fertility and productivity in eolian sandy soils on the Northeast China Plain.

## Data availability statement

Raw sequencing data were deposited in the NCBI Sequence Read Archive under accession number PRJNA1111948. The data of SOC, soil nutrients content and maize yields can be found in the article/supplementary material.

## Author contributions

Y-mL: Data curation, Funding acquisition, Investigation, Methodology, Writing – original draft. Y-mW: Writing – review & editing, Data curation, Investigation. G-wQ: Writing – review & editing. H-jY: Writing – review & editing. F-mL: Writing – review & editing. G-lW: Funding acquisition, Supervision, Writing – review & editing. YD: Funding acquisition, Investigation, Methodology, Supervision, Writing – original draft, Writing – review & editing.
