# Supplementary material for: Conservation tillage facilitates the accumulation of soil organic carbon fractions by affecting the microbial community in an eolian sandy soil
Source: Front Microbiol. 2024 May 31;15:1394179. doi: 10.3389/fmicb.2024.1394179 (PMC11176501; doi:10.3389/fmicb.2024.1394179)
Supplement: Supplementary file 1 [file Data_Sheet_1.docx]

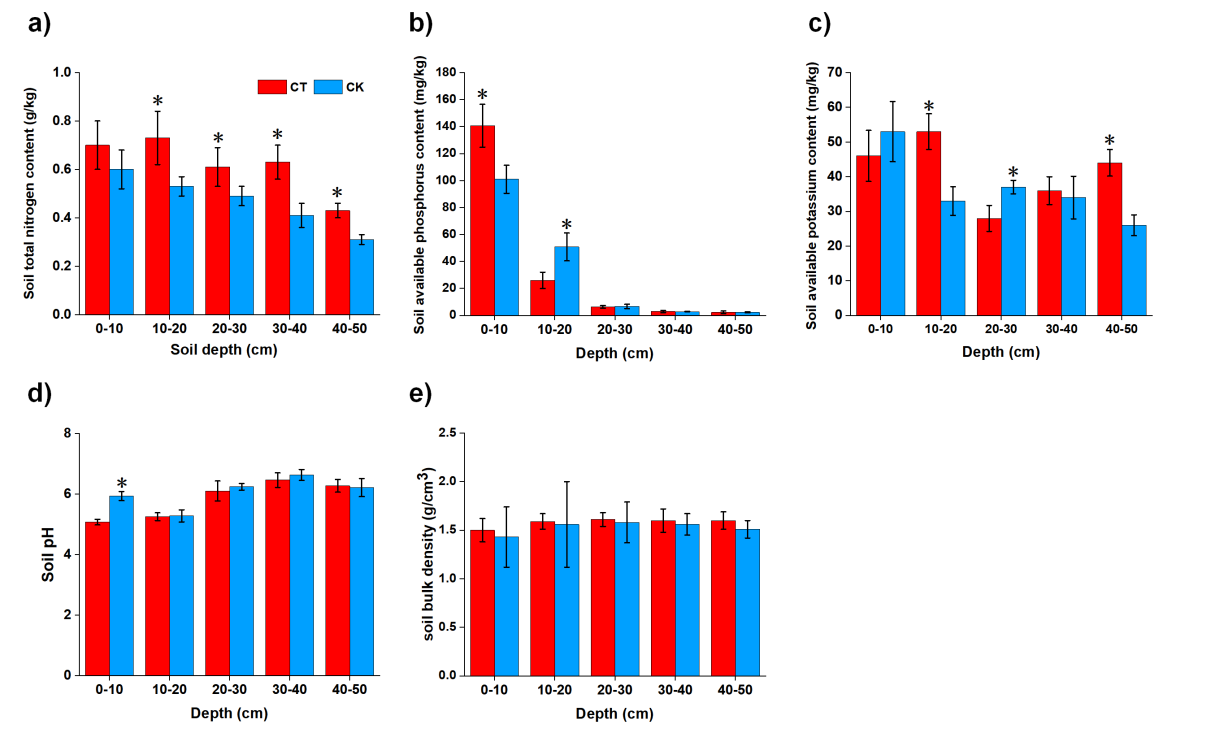


[Supplementary](https://www.frontiersin.org/journals/microbiology/articles/10.3389/fmicb.2024.1333526/full#SM1) Fig S1 The soil total nitrogen (a), available phosphorus (b), and available potassium (c) contents as well as the soil pH (d) and bulk density (e) at 0-50 cm soil depths under different tillage practices. *: *P* < 0.05; CT: conservation tillage; CK: traditional tillage.

[Supplementary](https://www.frontiersin.org/journals/microbiology/articles/10.3389/fmicb.2024.1333526/full#SM1) Fig S2 Maize yields under different tillage practices during 2019-2022. *: *P* < 0.05; CT: conservation tillage; CK: traditional tillage.


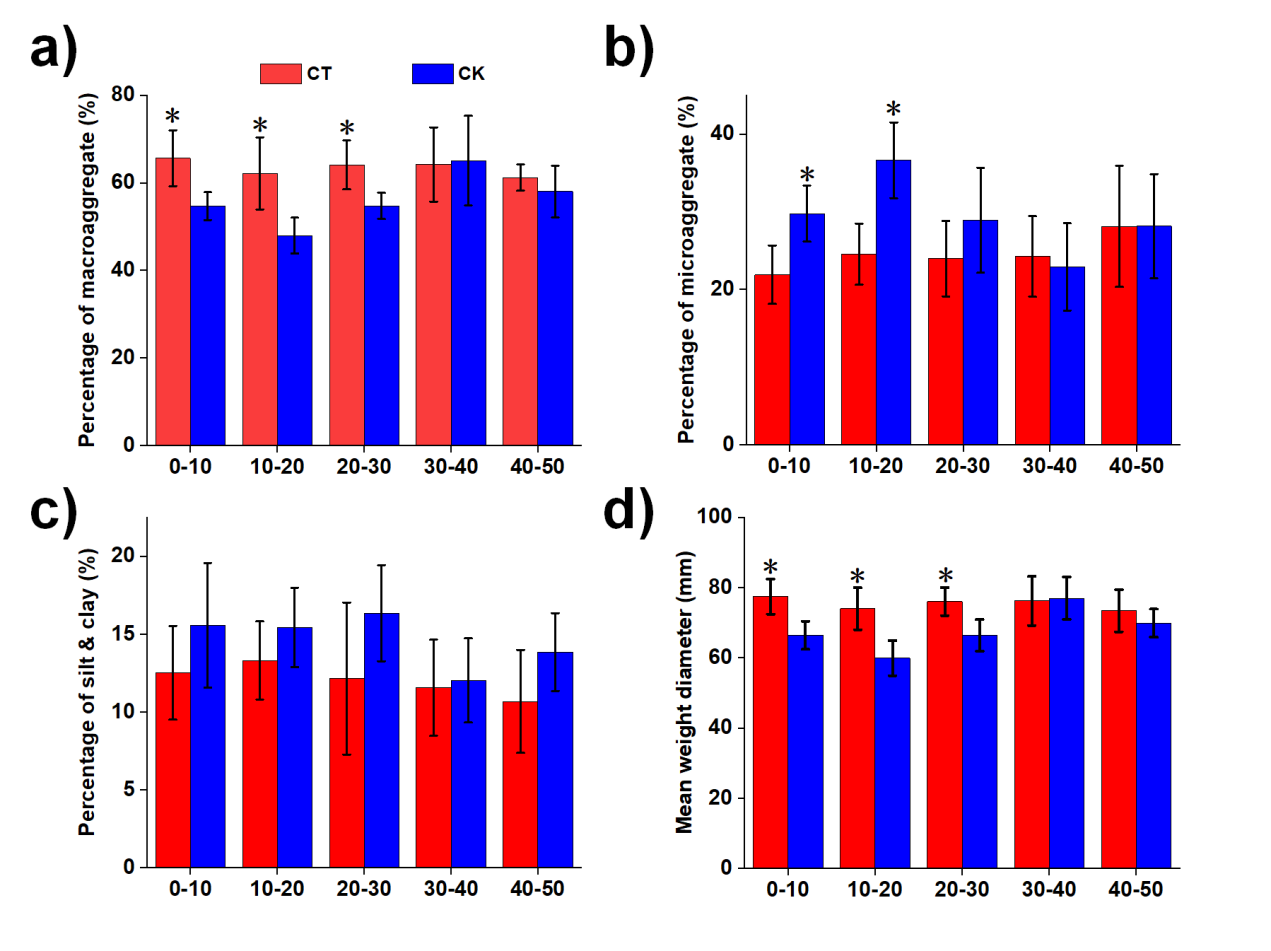


[Supplementary](https://www.frontiersin.org/journals/microbiology/articles/10.3389/fmicb.2024.1333526/full#SM1) Fig S3 The percentage of macroaggregates (a), microaggregates (b) and silt and clay (c), as well as the mean weight diameter (d), under the different tillage practices.


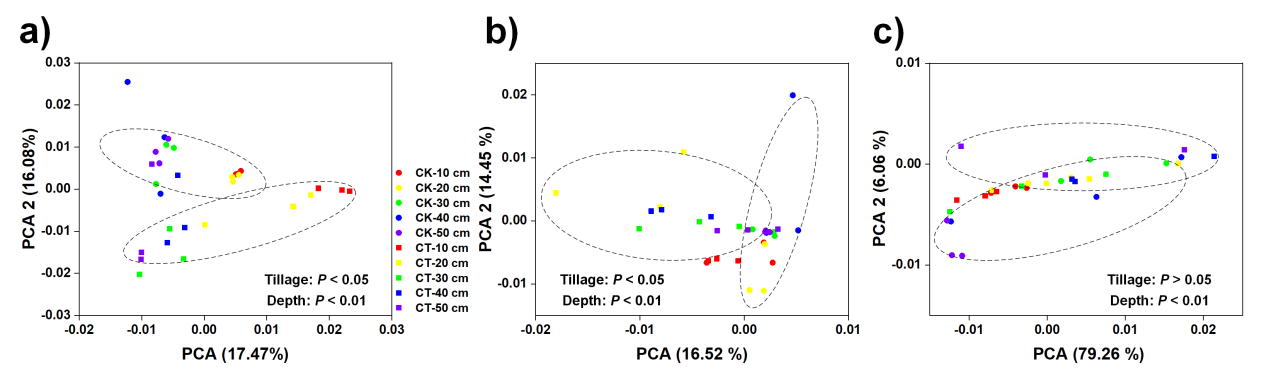


[Supplementary](https://www.frontiersin.org/journals/microbiology/articles/10.3389/fmicb.2024.1333526/full#SM1) Fig S4 Principal component analysis (PCA) showing the changes in the bacterial (a), fungal (b) and protistan (c) communities under the different tillage practices. The confidence interval represents the differences between tillage treatments. The effects of tillage practices and soil depth on the microbial community were examined via ANOSIM. *: *P* < 0.05; **: *P* < 0.01; CT: conservation tillage; CK: traditional tillage.


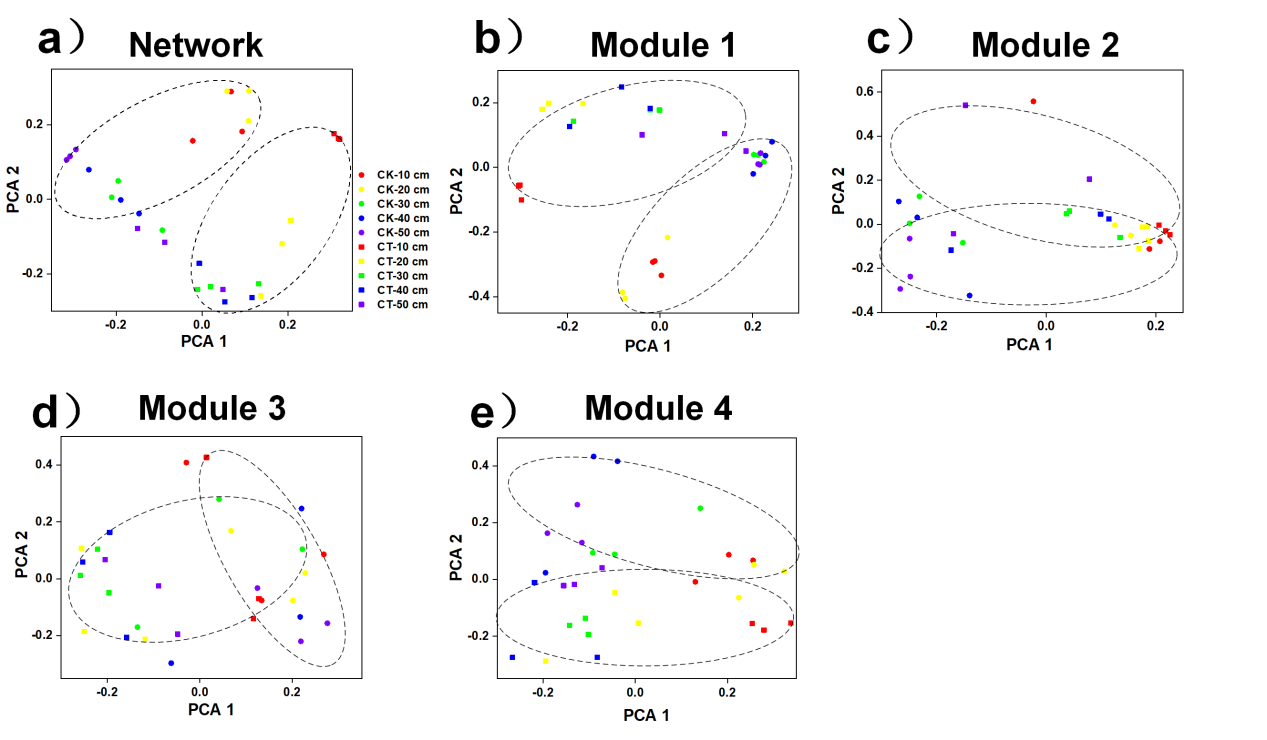


[Supplementary](https://www.frontiersin.org/journals/microbiology/articles/10.3389/fmicb.2024.1333526/full#SM1) Fig S5 Principal component analysis (PCA) showing the changes in the microbial community (a) and module 1-4 community (b-e) under the different tillage practices. CT: conservation tillage; CK: traditional tillage. The confidence interval represents the differences between tillage treatments.


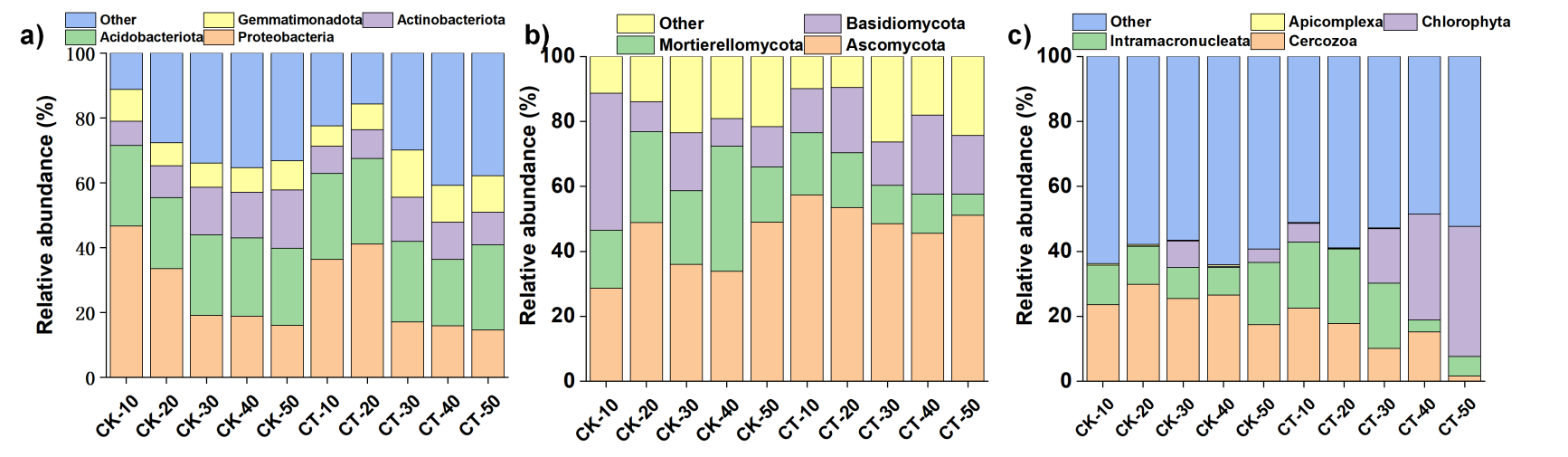
[Supplementary](https://www.frontiersin.org/journals/microbiology/articles/10.3389/fmicb.2024.1333526/full#SM1) Fig S6 Soil microbial community compositions of bacteria (a), fungi (b) and protist (c) under different tillage practices and depths at phylum scale.

[Supplementary](https://www.frontiersin.org/journals/microbiology/articles/10.3389/fmicb.2024.1333526/full#SM1) Table S1 Information on the selected keystone species

| ID | Kingdom | Phylum | Family | Module |
| --- | --- | --- | --- | --- |
| BASV17140 | Bacteria | Myxococcota | Myxococcaceae | 1 |
| BASV7998 | Bacteria | Actinobacteriota | Gaiellaceae | 1 |
| BASV174 | Bacteria | —— | —— | 1 |
| BASV7693 | Bacteria | Proteobacteria | Rhizobiales_Incertae_Sedis | 1 |
| BASV13129 | Bacteria | Chloroflexi | —— | 1 |
| BASV3331 | Bacteria | Proteobacteria | Xanthobacteraceae | 1 |
| BASV127 | Bacteria | Proteobacteria | Caulobacteraceae | 3 |
| BASV21184 | Bacteria | Acidobacteriota | Vicinamibacteraceae | other |
| BASV3531 | Bacteria | Verrucomicrobiota | Chthoniobacteraceae | 2 |
| BASV149 | Bacteria | Proteobacteria | Sphingomonadaceae | 1 |
| BASV11161 | Bacteria | Proteobacteria | Reyranellaceae | 1 |
| BASV8256 | Bacteria | Proteobacteria | —— | 1 |
| BASV408 | Bacteria | Proteobacteria | Rhizobiales_Incertae_Sedis | 3 |
| FASV95 | Fungi | Olpidiomycota | Olpidiaceae | 1 |
| FASV3791 | Fungi | Mortierellomycota | Mortierellaceae | 2 |
| FASV973 | Fungi | Basidiomycota | —— | 1 |
| FASV2452 | Fungi | Glomeromycota | Diversisporaceae | other |
| FASV391 | Fungi | Ascomycota | Pseudogymnoascus | 1 |
| FASV945 | Fungi | Ascomycota | Cephalotrichum | 1 |
| FASV1209 | Fungi | Ascomycota | Hypocreaceae | other |
| FASV1736 | Fungi | Ascomycota | Lasiosphaeriaceae | other |
| FASV946 | Fungi | Ascomycota | Herpotrichiellaceae | 1 |
| FASV3646 | Fungi | Ascomycota | Dipodascaceae | other |
| FASV2769 | Fungi | Chytridiomycota | —— | other |
| PASV45 | Protist | Cercozoa | —— | 1 |
| PASV266 | Protist | —— | —— | 1 |
| PASV2406 | Protist | Intramacronucleata | Chilodonellidae | other |
| PASV17 | Protist | Chlorophyta | —— | 1 |
| PASV1440 | Protist | —— | —— | 1 |
| PASV175 | Protist | Cercozoa | Heteromitidae | 1 |

Note: BASV: bacterial ASV; FASV: fungal ASV; PASV: protistan ASV.


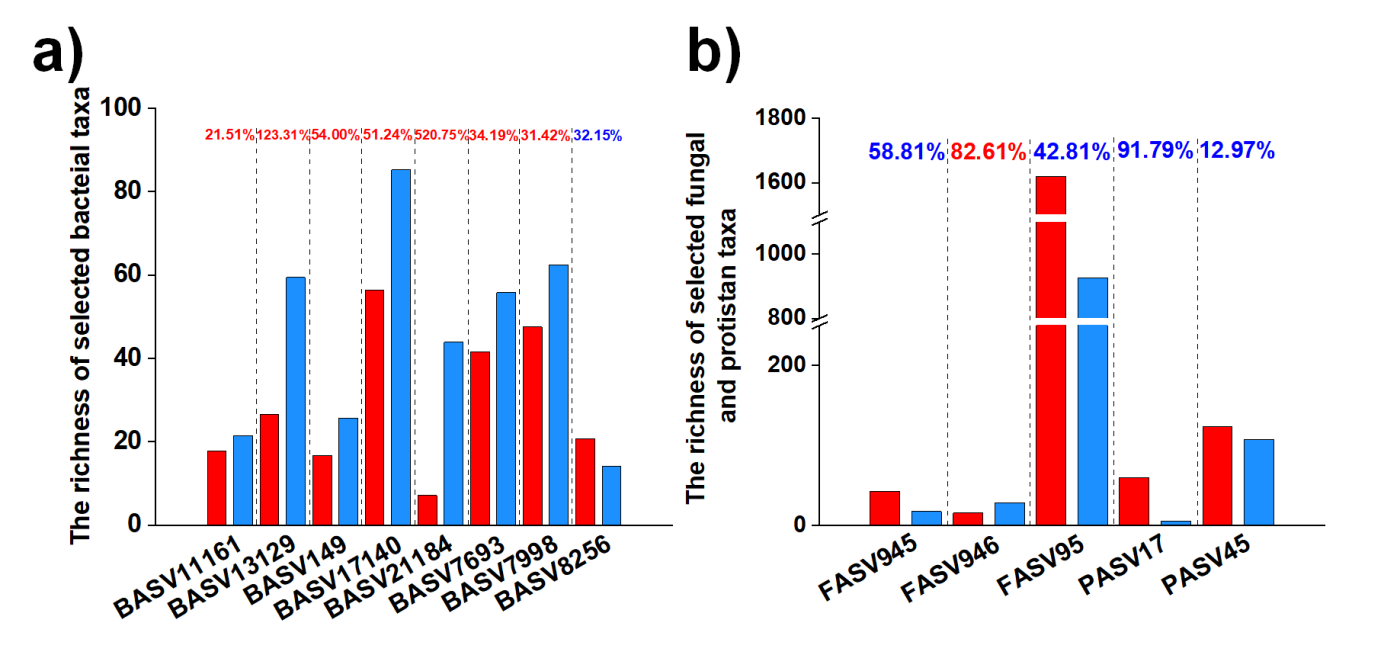


[Supplementary](https://www.frontiersin.org/journals/microbiology/articles/10.3389/fmicb.2024.1333526/full#SM1) Fig S7 The richness of selected keystone bacterial (a) as well as fungal and protist (b) species under different tillage practices. The numbers above the histogram indicate the changes in microbial taxon richness. The red number indicates a negative effect compared with that of CK, while the blue number indicates a positive effect. BASV: bacterial ASV; FASV: fungal ASV; PASV: protistan ASV.
